# Plant diversity and ethnobotanical importance of home gardens in Ghana’s middle belt: a cross-sectional survey of the Sunyani municipality

**DOI:** 10.1186/s13002-023-00632-1

**Published:** 2023-12-13

**Authors:** Bismark Ofosu-Bamfo, Daniel Yawson, Kwame Baffour Asare, Vanessa Ohui Dadeboe, Isaac Kojo Buabeng, Justice Aggrey, Dery Aaron Dapillah, David Kojo Boateng, Emmanuel Offe, Thomas Abudu Alhassan

**Affiliations:** 1 Department of Biological Sciences, School of Science, University of Energy and Natural Resources, Sunyani, Ghana; 2 Centre for Research in Applied Biology, School of Science, University of Energy and Natural Resources, Sunyani, Ghana; 3 The Ridge School (1966), P. O. Box 1659, Kumasi, Ghana

**Keywords:** Food security, Sustainable agriculture, Sustainable food, Sustainable Development Goal 2 (SDG 2)

## Abstract

**Background:**

Home gardens are a species-rich socioecological system with a diverse range of cultivated and naturally occurring plants with the potential to make contributions to address sustainable food, biodiversity and climate crisis. However, there is a dearth of information on the socio-demographic profile of home gardeners and the importance of home gardens to ethnobotany, food security and biodiversity. Therefore, the study aimed to assess the socio-demographic profile of home gardeners in the Sunyani municipality as a case in point for the middle belt of Ghana and to evaluate the diversity of plants in home gardens and their ethnobotanical importance.

**Methods:**

A total of 12 suburbs were selected from three subzones in the Sunyani municipality. In each suburb, 25% of households were randomly selected and if they had a home garden, one adult in the house was interviewed. A list of all plants in the home garden and their uses was obtained from respondents. A Chi-square test was used to assess the distribution of home gardeners among various socio-demographic categories, and binomial logistic regression was employed to determine links between socio-demography and home garden attributes. The ethnobotany R package was used to evaluate the ethnobotanical importance of plants in home gardens.

**Results:**

A total of 186 respondents were recruited for this study, 79 being females and 107 males. A total of 79 plant species were also identified belonging to 70 genera and 40 families. Trees were the most common plant life form in home gardens, followed by shrubs, herbs, vines, grasses and lianas. Ethnobotanical indices revealed the most important plants in home gardens to be staples, food supplements and medicinal plants. These were *Musa paradisiaca*, *Caripa pabaya*, *Xanthosoma sagittifolium*, *Manihot utilisima* and *Mangifera indica*, *Moringa oleifera*, *Citrus sinensis*, *Capsicum frutescens*, *Taraxacum officinale*, *Solanum aethiopicum*, *Cocos nucifera*, *Solanum torvum*, *Persea americana*, *Dioscorea alata* and *Elaeis guineensis*.

**Conclusion:**

Plants used as staples, food supplements and medicinal purposes emerged as the most culturally relevant scoring high on all ethnobotanical indices. Home gardens present an opportunity to address food security and nutrition needs of households and communities.

**Supplementary Information:**

The online version contains supplementary material available at 10.1186/s13002-023-00632-1.

## Background

Home gardens are species-rich socio-agro-ecological systems, usually small, but serving as repositories for diverse cultivated, enhanced and tolerated plant species while also contributing to in situ conservation of plant resources [1–4]. Home gardens hold immense potential in helping countries attain food security under Sustainable Development Goal 2: end hunger, achieve food security and improved nutrition and promote sustainable agriculture. This is because, home gardens can guarantee the four dimensions of food security, which are access, availability, use and sustainability [5]. Home gardens are easily accessible to households who often own them and attend to them. As such, food crops in home gardens would be available for use and can guarantee a sustainable supply of a family’s nutritional and food supply needs.

Knowledge of home gardens is relevant to determine how they can contribute to attaining some of the targets set by Ghana under SDG 2 [6]. Key among these targets are (1) doubling agricultural productivity and incomes of small-scale food producers, in particular women, indigenous peoples and family farmers, and (2) ensuring sustainable food production systems, implementing resilient agricultural practices to increase productivity and production, maintain ecosystems and strengthen capacity for adaptation to climate change, extreme weather, drought, flooding and other disasters and that progressively improve land and soil quality [6]. Additionally, home gardens can contribute to the government of Ghana’s key intervention on SDG 2 to promote the production and utilization of locally grown and nutrient-rich food which can help address the significant challenges that remain in achieving SDG 2 [7, 8]. Home gardens or backyard farming form part of recommendations made by the Civil Society Organizations Platform on SDGs in Ghana and the UN Communications Group in Ghana (The Sustainable Development Goals in Ghana, [9]. In Ghana, home gardens are recognized as a surviving food security strategy in the ecologically fragile northern regions [10]. It has also been identified as a means for in situ conservation of plant genetic resources [2].

The highly diverse plant species in home gardens are used in an array of ways such as food, spices, stimulants, decoration, medicines, beverages, fodder and shelter [11]. Their significance is extensively recognized in several domains including *inter alia*, food and nutritional security, medicine and health, poverty alleviation, biodiversity conservation, carbon sequestration, ecosystem service provisioning and socio-cultural preservation [12, 13]. Home gardens' vegetation structure, composition, function and diversity are influenced by socioeconomic considerations as well as the cultural values and interests of the people who manage them [14, 15]. Since home gardens reflect people’s socio-cultural values and traditional knowledge, their study provides an extensive understanding of ethnobotany [15, 16]. Thus, a dearth of information on home gardens means that indigenous botanical knowledge is likely in peril unless urgently documented [4, 17].

Notwithstanding the above-stated relevance, a comprehensive study of home gardens, their diversity and ethnobotany is generally lacking [18] and the case for a lower middle-income country such as Ghana is no different [19]. Therefore, it is imperative to capture and document this knowledge before it is completely lost. This would provide insight into the functioning of home gardens and provide essential information for formulating policies that are ecologically and socio-economically appropriate for sustainable and resilient agriculture and the maintenance of biodiversity in both urban and rural areas [20, 21]. Based on studies on home gardens, governments and nonstate actors will be better informed on target groups for financial and technological interventions to set up or scale up their involvement in home gardens. Thus, the specific aims of this study were to assess the socio-demographic profile of home gardeners in the Sunyani municipality as a case in point for the middle belt of Ghana and to evaluate the diversity of plants in home gardens and their ethnobotanical importance.

## Methods

### Description of the study area

**Fig. 1:**
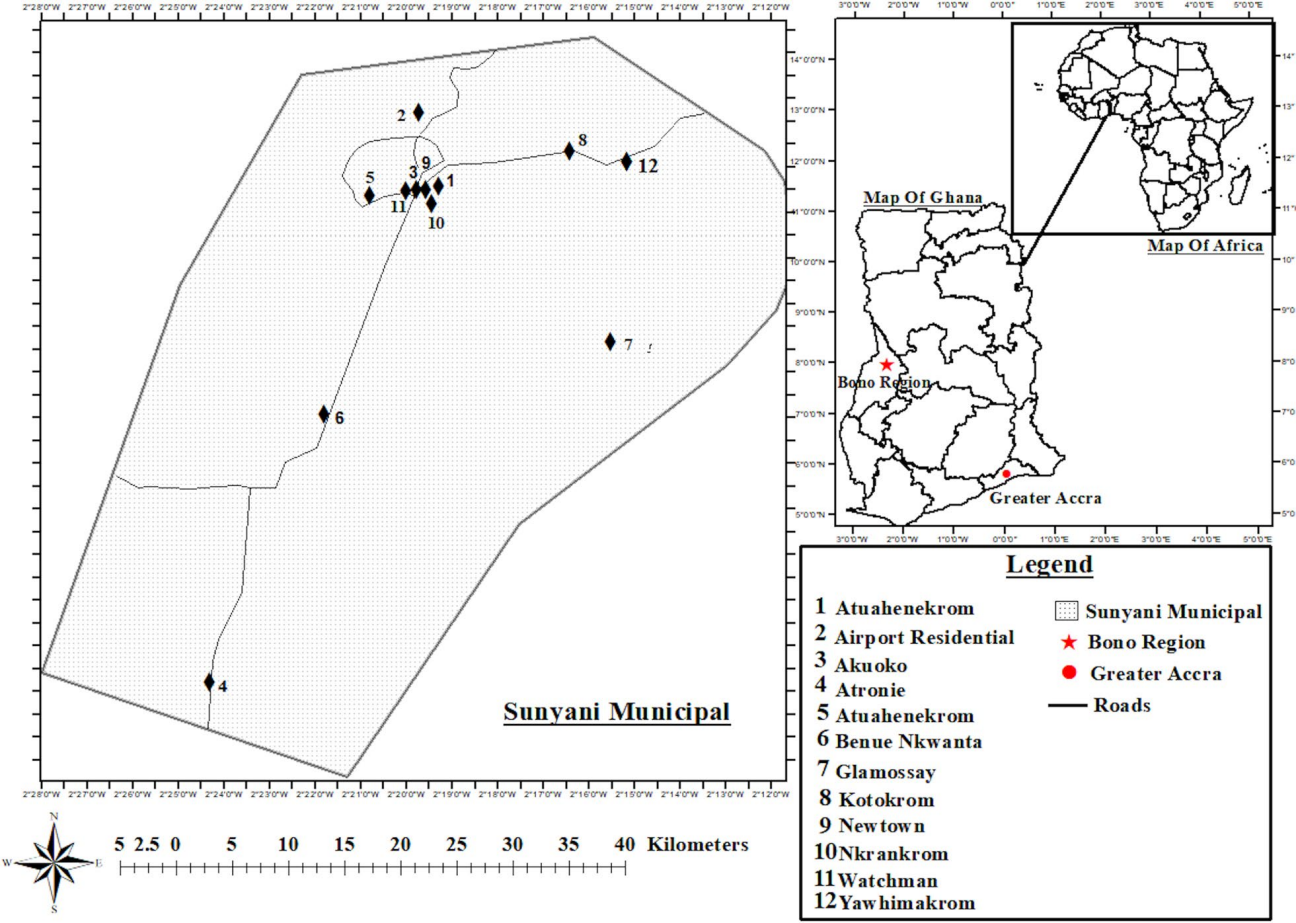
Map of study area

The fieldwork was conducted in Sunyani which is located in the Bono Region, between the latitudes of 7° 20′ N and 7° 05′ N and the longitudes of 2° 30′ W and 2° 10′ W. Sunyani Municipal, one of the indigenous towns of the Bono ethnic group, has a population of 193,595 people (49.78% males and 50.22% females), according to the 2021 Ghana Population and Housing Census projection Ghana Statistical Service [22]. The municipality of Sunyani is within Ghana's moist semi-deciduous forest zone. Monthly temperatures range from 23 to 33 °C, with August having the lowest temperatures and March and April having the highest. The district has a two-season rainfall pattern, with the main rainy season lasting from March to September and the minor wet season lasting from October to December [23]. During the wet season, the optimum relative humidity for lush vegetative development is between 75 and 80% and below 70% during the dry season. Twelve communities were selected from three administrative councils of the Sunyani municipality. The subzones and communities were Sunyani (Akuoko, Ahenboboano, Newtown, Airport Residential Area), Atronie (Atronie, Atuahenekrom, Benue Nkwanta, Watchman) and Abesim (Yawhimakrom, Kotokrom, Nkrankrom, Glamosay) (Fig. 1).

### Study design and data collection

A cross-sectional survey was employed in which individuals who owned a home garden in the municipality were selected and interviewed. Suburbs were selected using a stratified random approach, whereas home gardeners in each suburb were identified using snowball sampling where a study participant directed data collectors to the next prospective participant who has a home garden. In each community, community leaders were informed about the study and study participants who were above 18 years old, had home garden in their homes and consented to the study were interviewed. Demographic data of home gardeners and a comprehensive list of all plants found within their home garden and their uses were obtained following the methods of Whitney et al. [21]. List of plants was based on free listing by respondents rather than a checklist from interviewers. Interviews were conducted in Twi and Bono local languages common to the study communities. Specimen of plants in each home garden were collected to aid in their identification to morphospecies level. Taxonomical identification was done with the aid of Hawthorne and Jongkind [24] and voucher specimen kept in the herbarium of the Department of Biological Sciences at the University of Energy and Natural Resources, Sunyani, Ghana.

### Data analysis

The distribution of respondents among socio-demographic profiles was evaluated using the Chi-square test (n outcomes) in the Jamovi software. Significant differences were determined at alpha value of 0.05 based on the Chi-square test statistic *χ*^2^ and degrees of freedom (*df*, number independent variables). A binomial logistic regression analysis was also carried out to determine the relationship between demographic variables and plant life forms as well as motivation for home gardening. We also analysed the data to find out if motivation for home gardening affected plant life forms present in the home garden. This was done in *R* using the *glm* function (family = binomial) from the stats package. The ethnobotanical indices were computed using the ethnobotany R package in R program. These indices were use report (UR), number of uses (NU), frequency of citation (FC), cultural importance (CI), relative importance (RI) and fidelity level [21, 25]. These indices based on counts and weighted values indicate the importance, popularity and usability of a particular species. Use report is a value that indicates the total number of uses for a species reported by all informants, while number of uses simply refers to the number of use categories reported for each plant. Frequency of citation is the number of times a species was mentioned by all respondents. Relative frequency of citation is determined as the contribution of each species to the total frequency of citations. The cultural importance index is a weighted value determined as use reports for a species per respondent, whereas relative importance index is determined for each species as an average value of the relative frequency of the species divided by the maximum relative frequency and the number of uses of that species divided by the maximum number of uses. Fidelity level measures the agreement of respondents on the use of a particular species [21, 25].

## Results

### Socio-demography of home gardening in Sunyani municipal assembly

A total of 186 respondents into home gardening were interviewed in the Sunyani municipality. A significantly higher proportion were males (57.5%), while the rest were females (42.5%; Table 1). The majority of respondents belonged to the age bracket 31–40 (27%) and 41–50 (25%). The remaining were in age groups 51–60 (18%), 18–30 (17%) and > 60 (13%). Most respondents were married (70%), and the rest were distributed among single, divorced and widowed categories. A great percentage of respondents had at least basic education (48%), followed by secondary education (23%) and tertiary education (13%), while 15% had no formal education. Seventy per cent (70%) of respondents earned a monthly income of less than 1000 cedis (1 USD = 10 cedis based on current average). This was followed by a few others (20%) in the 1000–2000 cedis income category and fewer still in the 2000–3000 and > 3000 cedis income categories. Farming (45%), trading (23%) and working in the civil service (20%) were the three leading forms of employment among respondents into home gardening in the Sunyani municipality. Three per cent (3%) of respondents were retired, whereas 5% were unemployed. Among home gardeners in the Sunyani municipality who were interviewed, 59% were indigenes of Sunyani, while 41% were non-indigenes. The non-indigene respondents were mainly from Ashanti (23 respondents), Frafra (7 respondents), Fante (7 respondents), Dagaati (5 respondents), Ewe (3 respondents), Sisala (3 respondents), Anufo, Konkomba, Gonja (2 respondents each). Respondents were predominantly Christians (88%) but included Muslims (10%) and other religious groups (2%).

**Table 1:** Socio-demographic data collected from 186 respondents

Level	Count	Proportion	χ^2^	*df*	*p* value
*Gender*					
Female	79	0.425	4.22	1	0.04
Male	107	0.575			
*Age bracket*					
18–30	31	0.167	13.9	4	0.008
31–40	51	0.274			
41–50	47	0.253			
51–60	33	0.177			
> 60	24	0.129			
*Marital status*					
Single	31	0.1667	205	3	< .001
Married	130	0.6989			
Divorced	10	0.0538			
Widowed	15	0.0806			
*Level of education*					
None	28	0.151	58.3	3	< .001
Basic	90	0.484			
Secondary	43	0.231			
Tertiary	25	0.134			
*Monthly income*					
< 1000	131	0.7043	218	3	< .001
1000–1900	37	0.1989			
2000–3000	16	0.086			
> 3000	2	0.0108			
*Occupation*					
Farmer	84	0.45161	259	7	< .001
Trader	42	0.22581			
Civil servant	37	0.19892			
Student	5	0.02688			
Mason	1	0.00538			
Cleaner	1	0.00538			
Retired	6	0.03226			
Unemployed	10	0.05376			
*Indigene status*					
Indigene	109	0.586	5.51	1	0.019
Non-indigene	77	0.414			
*Religion*					
Christian	163	0.8763	249	2	< .001
Muslim	19	0.1022			
Other	4	0.0215			

The majority of respondents have been home gardeners for about 1–5 years (53%). The remaining were distributed among the 6–10 years (24%) and more than 10 years (23%) categories. The purpose for maintaining home gardens by respondents was mainly for food as indicated by 76% of respondents (Table 2). Other common reasons for keeping home gardens were for recreational purposes (14.5%; shade, beautification), medicinal plants (13%), demarcating boundaries (8%) and income generation (2%). A significantly high number of respondents had trees in their home gardens (94%) compared to those who did not have trees. Respondents who had shrubs in their gardens were similar to those who did not (50% each). Herbs (19%), vines (11%), grasses (10%) and lianas (4%) were less frequent in home gardens of respondents.

**Table 2:** Purpose for home gardens and major plant life forms used

Attribute	Count	Proportion	χ^2^	*df*	*p* value
*Length of time in home gardening*					
1–5 years	99	0.532	33.2	2	< .001
6–10 years	45	0.242			
> 10 years	42	0.226			
*Purpose of home gardening*					
Boundary					
No	172	0.9247	134	1	< .001
Yes	14	0.0753			
Recreation					
No	159	0.855	93.7	1	< .001
Yes	27	0.145			
Food					
No	44	0.237	51.6	1	< .001
Yes	142	0.763			
Medicine					
No	162	0.871	102	1	< .001
Yes	24	0.129			
Income					
No	182	0.9785	170	1	< .001
Yes	4	0.0215			
*Plant life form in home garden*					
Grass					
No	167	0.898	118	1	< .001
Yes	19	0.102			
Vine					
No	165	0.887	111	1	< .001
Yes	21	0.113			
Tree					
No	11	0.0591	145	1	< .001
Yes	175	0.9409			
Shrub					
No	93	0.5	0	1	1
Yes	93	0.5			
Liana					
No	178	0.957	155	1	< .001
Yes	8	0.043			
Herb					
No	150	0.806	69.9	1	< .001
Yes	36	0.194			

Respondents level of education (no education: estimate = 1.84, standard error = 0.86, *z* value = 2.13, *p* value = 0.033 and tertiary education: estimate = 2.87, standard error = 1.43, *z* value = 2.00, *p* value = 0.045) significantly favoured the odds having grass in home gardens. The odds of vines present in home gardens of respondents were significantly enhanced by income level 2000–3000 cedis (estimate = 2.91, standard error = 1.11, *z* value = 2.61, *p* value = 0.009). The odds of shrubs in respondents' home gardens were significantly affected by marital status (widowed: estimate = 2.77, standard error = 1.08, *z* value = 2.56, *p* value = 0.011) and level of education (secondary: estimate = 1.59, standard error = 5.80, *z* value = 2.74, *p* value = 0.006; tertiary: estimate = 1.88, standard error = 0.91, z value = 2.07, *p* value = 0.038). Age categories (31–40: estimate =  − 2.46, standard error = 1.01, *z* value =  − 2.42, *p* value = 0.015; 41–50: estimate =  − 3.04, standard error = 1.11, *z* value =  − 2.75, *p* value = 0.006; 51–60: estimate =  − 2.82, standard error = 1.14, *z* value =  − 2.48, *p* value = 0.013) and being male (estimate =  − 1.28, standard error = 0.59, *z* value =  − 2.19, *p* value = 0.028) significantly decreased the odds of herbs in home gardens. None of the socio-demographic variables significantly affected the odds of trees and lianas present in home gardens of respondents. Socio-demographic variables influenced some of the motivation of respondents for keeping home gardens. Respondents’ occupation as students (estimate = 3.64, standard error = 1.84, *z* value = 1.98, *p* value = 0.048) significantly increased the odds of recreation as the reason for keeping home gardens. The odds of food as the reason for keeping home gardens was improved by level of education (secondary: (estimate =  − 1.28, standard error = 0.59, *z* value =  − 2.19, *p* value = 0.028) but decreased by non-indigene ethnicity status (estimate =  − 1.28, standard error = 0.59, *z* value =  − 2.19, *p* value = 0.028). Non-indigene ethnicity status also decreased the odds of keeping home gardens for medicinal purposes. Boundary demarcation and income generation were not affected by socio-demographic variables. The odds of plant life forms present in home gardens were in some instances affected by the reason for keeping home gardens. The presence of grass was significantly affected by medicine as the reason for keeping home gardens (estimate = 1.26, standard error = 0.62, *z* value = 2.02, *p* value = 0.043). The presence of shrubs was affected by both boundary (estimate =  − 2.48, standard error = 1.03, *z* value =  − 2.42, *p* value = 0.016) and recreation (estimate = 1.86, standard error = 0.82, *z* value = 2.27, *p* value = 0.024).

### Plant diversity and ethnobotanical importance of home gardens

**Fig. 2:**
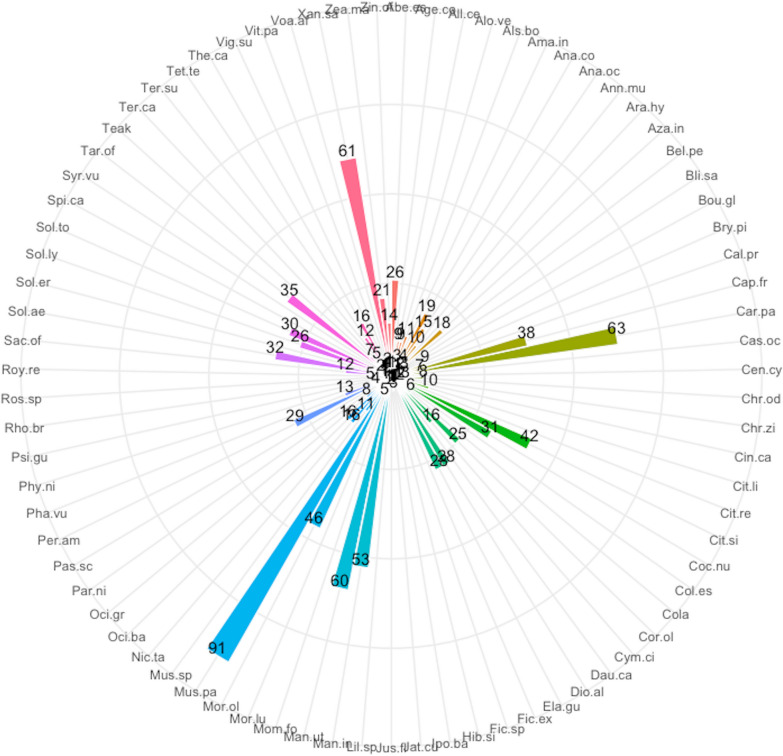
Use report of species in home gardens in the Sunyani municipality (full species names in Additional file 1: Appendix 1)

**Fig. 3:**
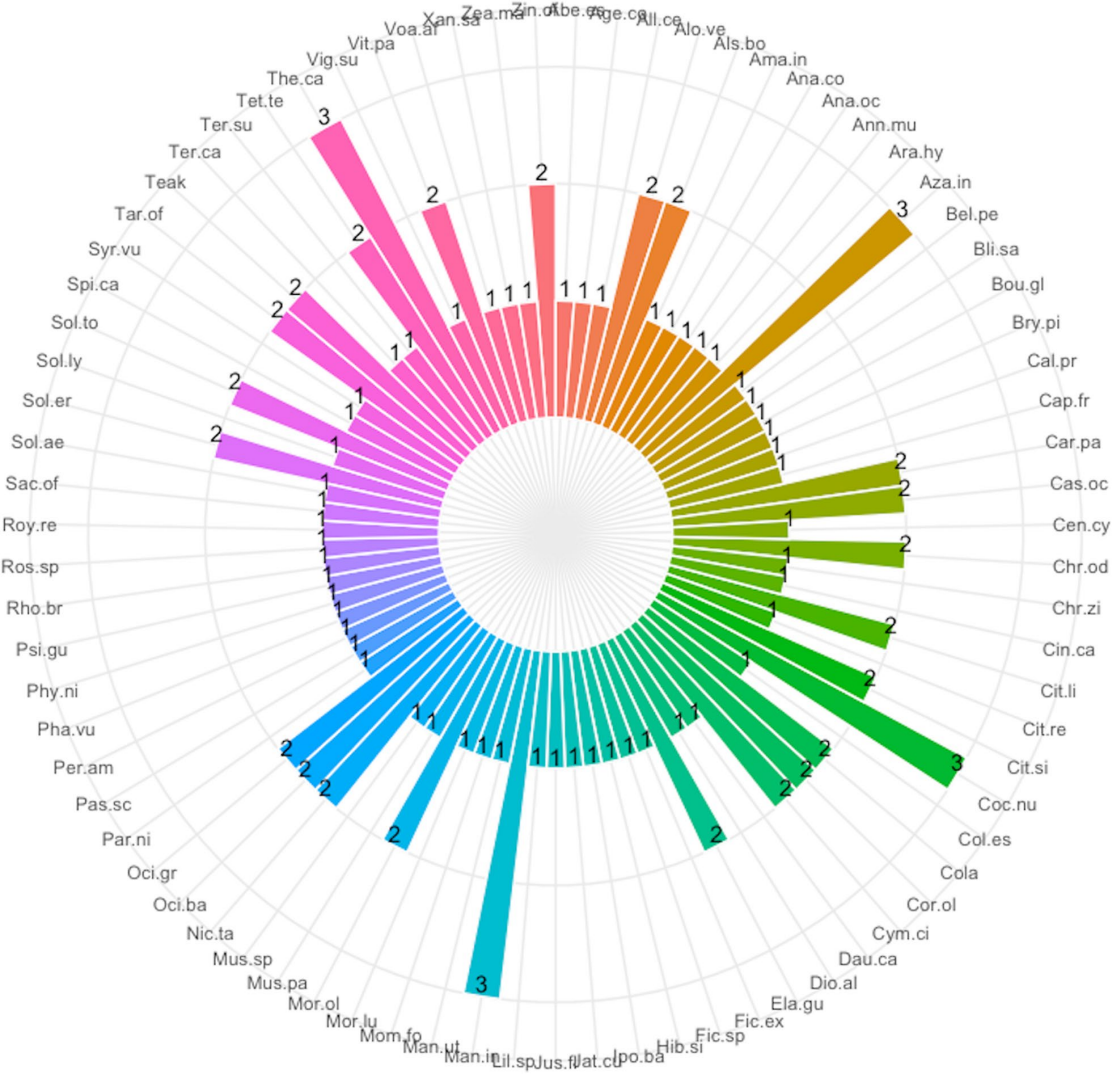
Number of uses of species in home gardens in the Sunyani municipality (full species names in Additional file 1: Appendix 1)

**Fig. 4:**
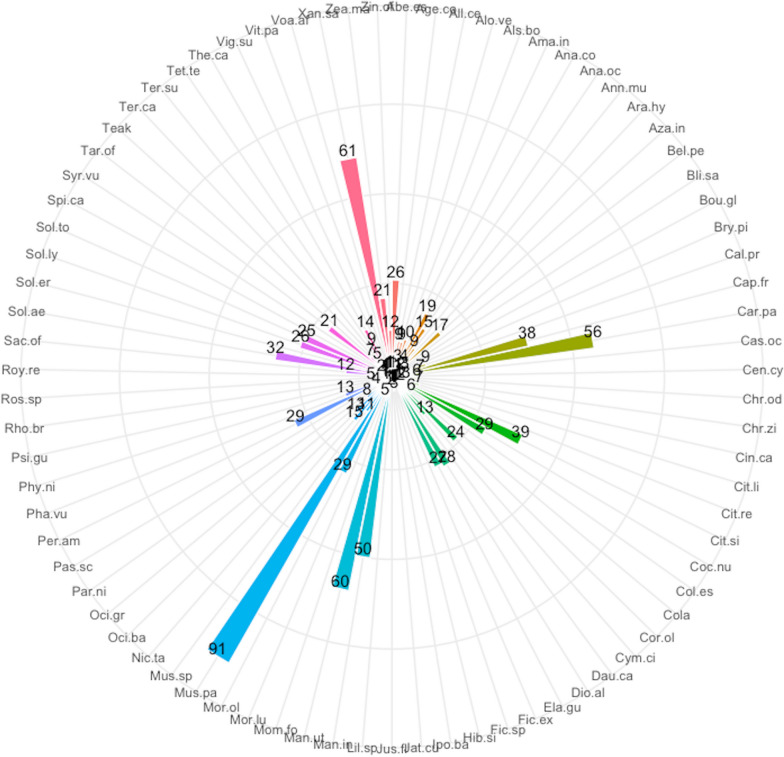
Frequency of citation of species in home gardens in the Sunyani municipality (full species names in Additional file 1: Appendix 1)

**Fig. 5:**
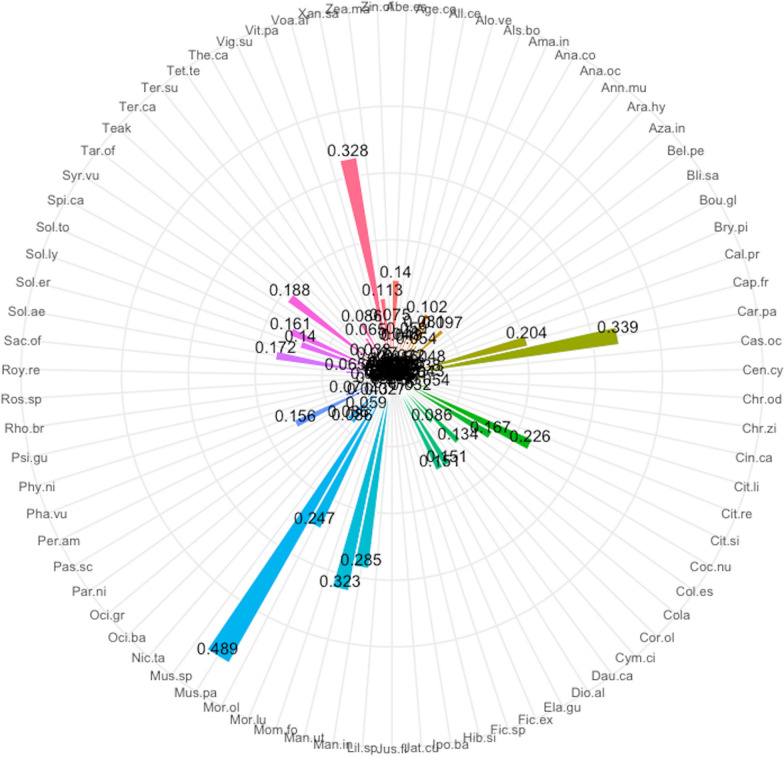
Cultural importance index of species in home gardens in the Sunyani municipality (full species names in Additional file 1: Appendix 1)

**Fig. 6:**
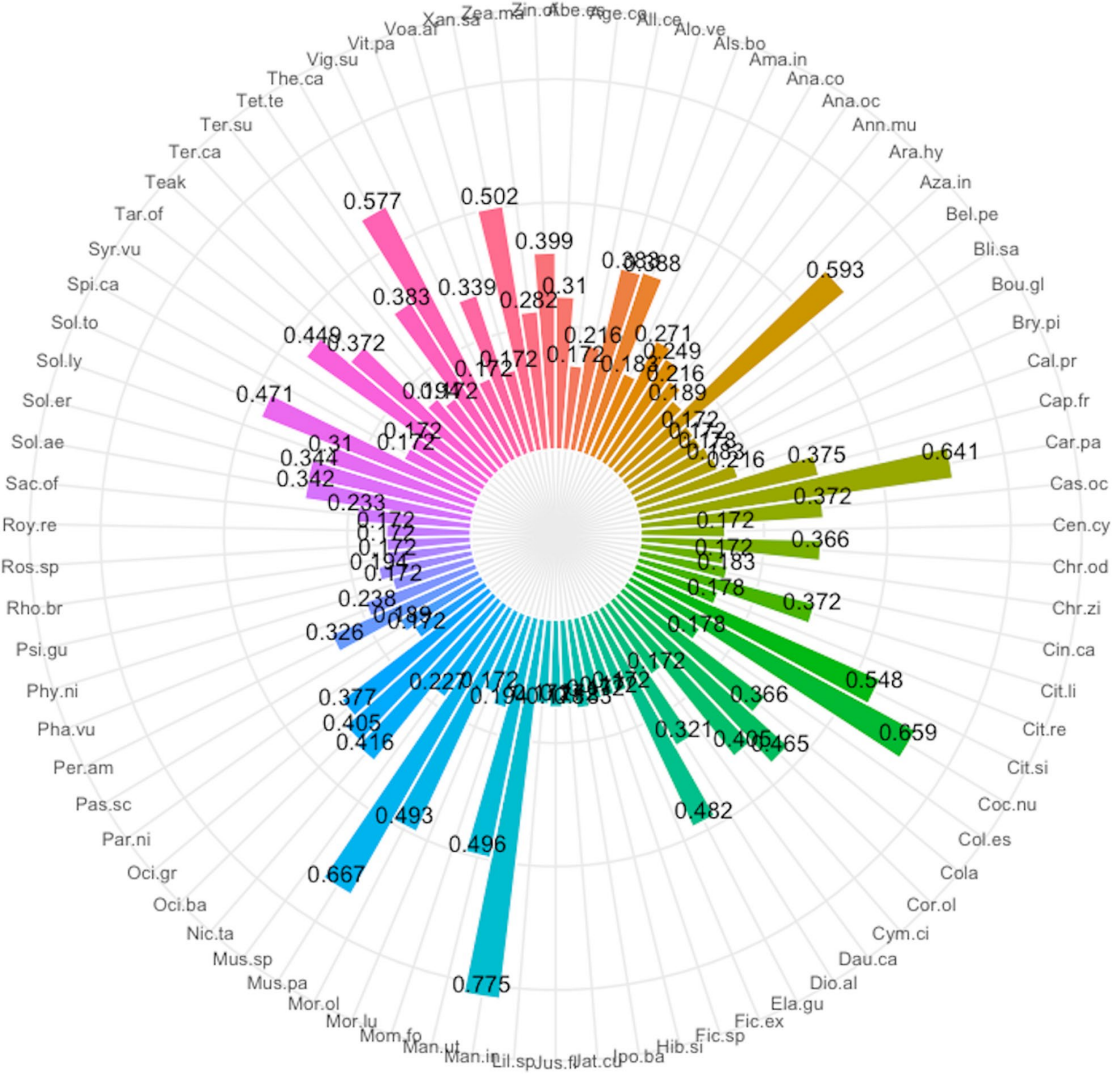
Relative importance index of species in home gardens in the Sunyani municipality (full species names in Additional file 1: Appendix 1)

**Fig. 7:**
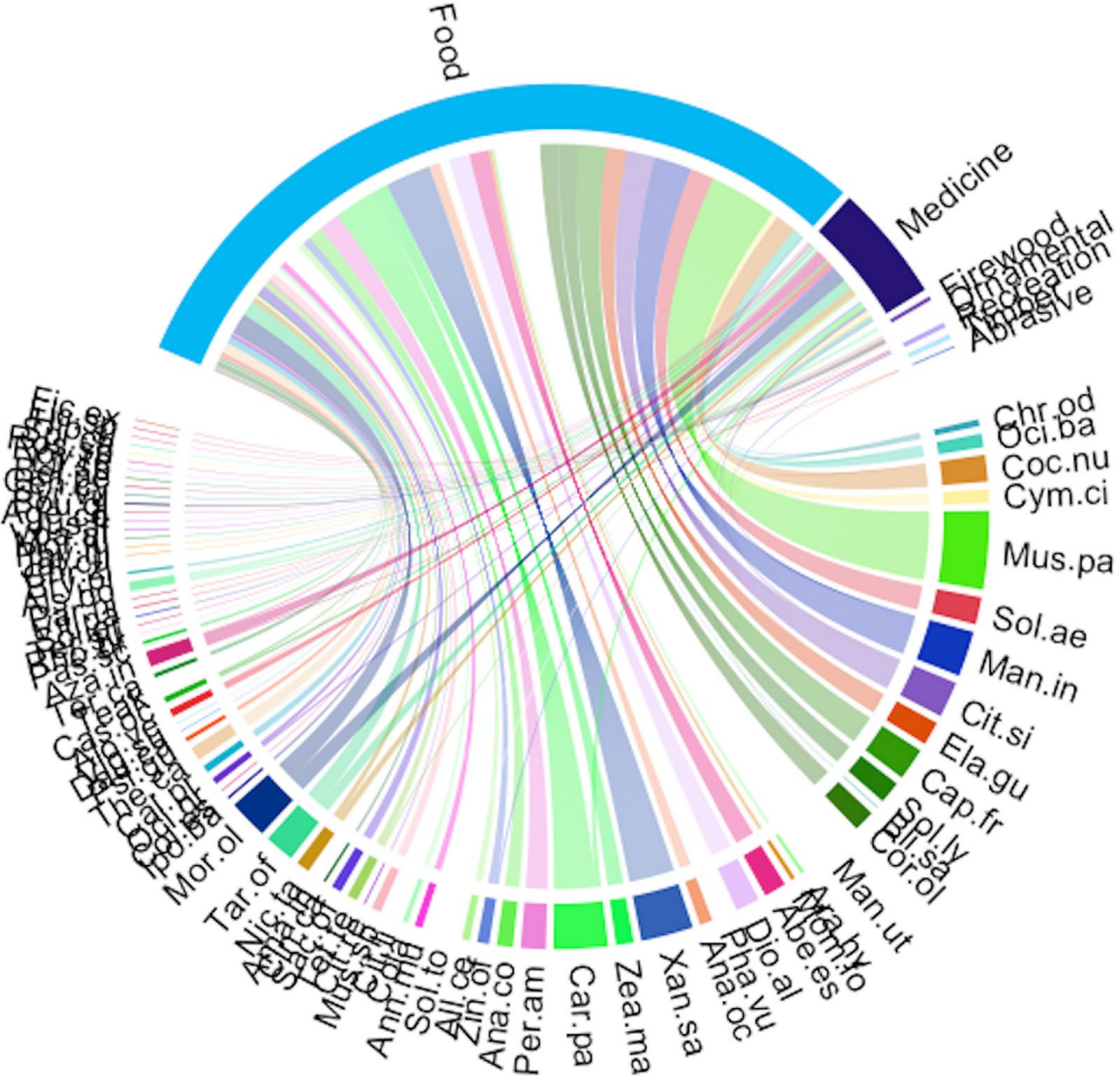
Chord plot of species and their contribution to use categories (full species names in Additional file 1: Appendix 1)

A total of 79 plant species were recorded in home gardens in the Sunyani municipality (Additional file 1: Appendix 1). These belonged to 40 families and 70 genera. The most common life forms were trees (30 species), followed by herbs (21 species), shrubs (18 species), vines (4 species), lianas (3 species) and grasses (3 species). Ethnobotanical analysis revealed *Musa paradisiaca* (91) had the highest use report. It was followed by *Carica papaya* (63), *Xanthosoma sagittifolium* (61), *Manihot utilisima* (60) and *Mangifera indica* (53) (Fig. 2). Others that were also highly ranked next to the top five (5) were *Moringa oleifera*, *Citrus sinensis*, *Capsicum frutescens*, *Taraxacum officinale*, *Solanum aethiopicum*, *Cocos nucifera*, *Solanum torvum*, *Persea americana*, *Dioscorea alata* and *Elaeis guineensis*. The same set of species had the highest number of uses, frequency of citation, cultural importance and relative importance index provided by respondents (Figs. 3, 4, 5, 6). The most common use category was food as shown in the chord plot (Fig. 7). Several species contributed to this use category, the principal among them being, *Musa paradisiaca*, *Manihot utilisima*, *Carica papaya*, *Xanthosoma sagittifolium*, *Mangifera indica* and *Citrus sinensis*. Medicinal uses were the next important use category of species found in home gardens. This was mainly accounted for by *Moringa oleifera*, *Azandiracta indica*, *Taraxacum officinale* and *Nicotiana tabacum*. High fidelity levels were recorded for food and ornamental uses of most species that occurred in home gardens (Additional file 1: Appendix 2). Forty-nine out of 58 times food was recorded as a use type, and it had a fidelity level score greater than 80. Medicinal use occurred 32 times and had 11 records of fidelity level score > 80. Ornamental uses had 9 out of 11 occurrences recording fidelity level > 80.

## Discussion

### Socio-demography of home gardening in Sunyani municipal assembly

Our study showed that more males were involved in home gardening in the Sunyani municipality than females and most respondents were also married. This indicates that most home gardening is led by men and occurs within family setups. Akrofi et al. [26] highlighted the significance of gendered roles in land and labour dynamics in the agricultural setting which is also reflected in this study. This was also confirmed by Bagson and Beyuo [10]. Married or dual-headed households (which are practically led by males) in their study planted more categories of crops and higher diversity of crop species compared to other households. This could be attributed to labour-intensive components of home gardening, such as land clearing, fencing and weeding which are traditionally viewed as the male tasks [10, 26]. The number of home gardeners generally decreased with increasing level of education and increasing level of income which suggests that any intervention that can potentially transform home gardens into income-generating hubs would be a huge poverty alleviation avenue [4]. We observed that most home gardeners in our study were farmers and traders who may be more motivated to keep a garden for supplemental income and for extra food to reduce household expenditure on food. For farmers, keeping a garden could also serve as a pastime activity or supplementary to a bigger and more distant farm. Traders may also keep home gardens to get extra items to sell from their gardens. This trend of decreasing interest in home gardens with increasing education and income was validated also by the few public sector workers who had home gardens in our study. Bolang and Osumanu [27] also noted a lack of interest by formal sector workers in Ghana to participate in home gardening. This trend needs to be reversed because employers in the formal sector could have access to credit facilities with which they can revamp urban agriculture to address employment and food security needs. It is also worth noting that most home gardeners were indigenes which means investment in home gardens can transform local economies, from rural to urban locations. While the study suggests most home gardeners were Christians and few were Muslims, we are of the view it is only a reflection of the demography of the wider population rather than a lack of interest on the part of any specific religious group [22].

The dominant age bracket of people in home gardening was 31–50 years. People within these categories are mostly strong for the physical demands of work in the garden including fencing, maintenance and weeding. Most respondents have been home gardeners 1–5-year and 6–10-year durations and only a few belonged to the more than 10-year duration. Respondents maintain home gardens mainly for food. Other common reasons were for recreation (shade, beautification), medicinal plants, demarcating boundaries and income generation. Almost all respondents had trees in their home gardens but only half of the respondents had shrubs and less than half had other life forms (herbs, vines, grasses, lianas). Home gardens could serve as an important avenue to improve tree cover in urban areas and make communities ecologically resilient to climate change impacts. However, for home gardens to provide agricultural resilience and food security, a lot more gardeners need to be encouraged to plant shrubs, herbs and vines which constitute most staple foods in the localities of the Sunyani municipality. Further analysis of the results showed that the choice of plant life forms in home gardens was influenced by education, income, marital status, age and gender as well as the reason for keeping home gardens (medicine, boundary setting and recreation). This establishes that the background of home gardeners and their motivation for home gardening can influence the plants that occur in home gardens. The motivation to keep home gardens in itself was also affected by occupation, education and indigene status of respondents.

### Plant diversity and ethnobotanical importance of home gardens

The species richness and various life forms recorded in home gardens in the Sunyani municipality show the potential of home gardens to contribute to addressing food security, biodiversity and climate crisis [4, 28]. The different life forms create a multilayered stratification within home gardens [15] which means they can potentially support a range of non-domestic fauna (e.g. birds, rodents, insects, invertebrates, reptiles, etc.) within the home garden ecosystem. These organisms when supported by home gardens will equally provide a wide range of ecosystem services including pollination, dispersal, pest control and soil quality improvement [28]. The Sunyani municipality being adjacent to the fragile dry semi-deciduous transition zone ecosystem needs to be well positioned to cushion these areas from adverse climate impact with as many as 31 important tree species recorded in home gardens. These trees also are an important source of food, vitamins and minerals and medicine. *Carica papaya* (pawpaw), *Mangifera indica* (mango), *Citrus sinensis* (orange), *Cocus nucifera* (coconut) and *Persea americana* (avocado) are important fruits that provide nutrient supplements [29–34].

*Carapa proceraCarica papaya*, for example, is a reliable source for vitamins (A, C and riboflavin) and minerals (calcium, iron, potassium), and fibres but low in calories. Medicinal uses for the plant previously reported include antibacterial, antihelminthic, antimalaria, birth control and blood pressure control [29]. *Cocus nucifera* also similarly has antibacterial properties and rich in minerals such as potassium, calcium, magnesium and phosphate [30] along with several peptides that have high antioxidant activity [32]. *Mangifera indica* is a major source of both macronutrients and micronutrients. Its macronutrient composition includes carbohydrates, proteins, amino acids and lipids whereas micronutrient components comprise vitamins A, E, K, C and B1, B2, B3, B5, B6 and B12 [34]. *Citrus sinensis* though notable for its rich ascorbic acid content and antioxidant activity [35, 36] has some medicinal uses including analgesic, anti-inflammatory, anthelmintic, antibacterial, antifungal and hypolipidemic properties [37]. *Persea americana* is rich in oils and bioactive compounds that enhances immune system and provides protection from oxidative damage [33]. *Moringa oleifera* (moringa) is a very common scientifically acclaimed food supplement which can be added to several meals at various stages of preparation. The content vitamin A, calcium, iron, vitamin C, potassium and protein in *Moringa oleifera* leaves exceeds most plants [38]. Medicinally, *Moringa oleifera* has been used for antidyslipidemic, anthelmintic, antihyperglycaemic, anti-inflammatory, antibacterial, antioxidant, hepatoprotective and neuroprotective purposes [38–40].

The contribution of herbs and shrubs to the species richness recorded in home gardens is a reflection of the life form of most Ghanaian staple foods which are sourced from herbs and shrubs [41]. The most important species identified in the home gardens of the Sunyani municipality are all used in households regularly for both nutritious and culturally important diets. *Musa paradisiaca* (plantain), *Manihot utilisima* (cassava), *Xanthosoma sagittifolium* (cocoyam) and *Dioscorea alata* (yam) are important for ampesi, individually and for fufu when *M. paradisiaca* and *Manihot utilisima* are combined (but *D. alata* is used for fufu standalone) [42–45]. Fufu is a common and an important cultural food in most Ghanaian homes but particularly for the Akans (including the Bono people indigenous to Sunyani) and several other tribes in Ghana [46]. *Musa paradisiaca* is rich in iron, potassium, vitamin A and carbohydrates and can be used ripe or unripe, fried or boiled, whereas *Manihot utilisima*, also rich in calcium, phosphorus, iron and vitamin C, is considered the most affordable source of carbohydrate in tropical Africa [47–49]. When used in another form to prepare cassava semolina (also known as “gari” [50], *Manihot utilisima* could be used as an important snack and also as main diet when used for “eba,” a local staple food made from mixing and turning gari in hot water [47, 50]. *Xanthosoma sagittifolium* is a staple used by over 400 million people across the tropics and subtropics. It contains high levels of zinc and potassium and can be used by cooking, frying or roasting, while its leaves are used for both soup and sauce [42, 51].

Herbs and shrubs are also important sources of plant-based medicine and nutrient supplements. *Taraxacum officinale* (dandelion) has many medicinal and nutritional uses attributed to its name, the most common being antioxidant, hepatoprotective, anti-inflammatory, anticancer, antifungal, antiviral and antidiabetic properties [52–54]. *Solanum torvum* (turkey berries) is locally prescribed for patients and pregnant women with anaemia or taken to prevent anaemia in Ghana and Ivory Coast [55, 56]. It is frequently used in palm nut soup prepared from *Elais guineensis* (oil palm) or combined with *Solanum aethiopicum* (garden eggs) in sauce that may be an accompaniment to ampesi, a meal prepared from *Musa paradisiaca*, *Manihot utilisima*, *Xanthosoma sagittifolium* or *Dioscorea alata*. *Capsicum frutescens* (pepper) is an important spice for several meals [57, 58]. These demonstrate the importance of home gardens in addressing malnutrition and food security needs which are common problems in the tropics and subtropics [4].

The most common use category of plants in home gardens was food contributed mainly by *Musa paradisiaca*, *Manihot utilisima*, *Carica papaya*, *Xanthosoma sagittifolium*, *Mangifera indica* and *Citrus sinensis*. This further reinforces the purpose for which most respondents maintain home gardens. Another important use of home garden plants was medicinal purposes which was also cited as a reason for maintaining gardens. Some medicinal plants found in home gardens in Sunyani were *Moringa oleifera*, *Azandiracta indica*, *Taraxacum officinale* and *Nicotiana tabacum*. Wakhidah et al. [15] also identified food and medicine as the most important use for home gardens in Indonesia. While uses for food had a high level of agreement among respondents, few medicinal uses had high fidelity levels. Respondents agreed largely to plants which were used for ornamental purposes. In general, ornamental and recreational uses of home gardens were poorly referenced by respondents although they also hold therapeutic potential. In the middle of the COVID-19 pandemic, Zhang et al. [59] reported home gardens served as “ecological medicine” for mental health and overall wellbeing [59]. From the forgoing, all a household’s nutrient requirements could be met in provisioning ecosystem services of a home garden. Saaka et al. [60] reported most households with a home garden where more than three times likely to improve their dietary habit to include more fruits and vegetables than households with no home garden, following 7 months of nutrition education for both groups. As such, ready availability of nutrient sources in home gardens is important to obtain nutrition requirements towards achieving SDG 2.

## Conclusion and implications

Our study shows that home gardens can potentially contribute to meeting food security and nutrient needs of households and communities, thereby inching us closer to achieving sustainable development goal 2. A number of opportunities remain for policy interventions from governments and development partners. For example, more women need to be encouraged to take up home gardening. Financial and technical support for home gardening could yield outcomes of improved livelihood for people at the lower end of income spectrum, most of whom were home gardeners in our study. Going forward, it will be useful to develop a nutritional profile index for home gardens as a way of measuring how well plant diversity within gardens meets nutritional deficits of households and communities. This will be a more effective way to determine the contribution of home gardens in addressing malnutrition and food security challenges.

### Supplementary Information

**Additional file 1.** Ethnobotany index.

## Data Availability

The datasets used and/or analysed during the current study are available from the corresponding author on reasonable request.

## Abbreviations

SDG: Sustainable Development Goals
UN: United Nations
GSS: Ghana statistical service
UR: Use report
NU: Number of uses
FC: Frequency of citation
CI: Cultural importance
RI: Relative importance
FL: Fidelity level
Df: Degree of Freedom
*P* value: Probability value
*χ*^2^: Chi-square test statistic
COVID-19: Corona Virus Disease 2019
